# Age-Dependent Inflammatory, Lipid, and Echocardiographic Phenotypes in Acute Myocardial Infarction: Associations with In-Hospital Adverse Clinical Events

**DOI:** 10.3390/biomedicines14051066

**Published:** 2026-05-08

**Authors:** Bogdan-Sorin Tudurachi, Larisa Anghel, Andreea Tudurachi, Radu Andy Sascău, Cristian Stătescu, Mircea Ovanez Balasanian, Nicoleta Dubei, Delia Șalaru

**Affiliations:** 1 Internal Medicine Department, “Grigore T. Popa” University of Medicine and Pharmacy, 700503 Iași, Romania; bogdan-sorin.tudurachi@d.umfiasi.ro (B.-S.T.); leonte.andreea@d.umfiasi.ro (A.T.); radu.sascau@umfiasi.ro (R.A.S.); cristian.statescu@umfiasi.ro (C.S.); ovanes718@yahoo.com (M.O.B.); nicoletadubei@yahoo.com (N.D.); deliasalaru@gmail.com (D.Ș.); 2 Cardiology Department, Cardiovascular Diseases Institute “Prof. Dr. George I. M. Georgescu”, 700503 Iași, Romania

**Keywords:** acute myocardial infarction, risk stratification, C-reactive protein, lipoprotein(a), neutrophil-to-lymphocyte ratio, systemic immune–inflammation index, left ventricular ejection fraction, global longitudinal strain

## Abstract

**Background:** Acute myocardial infarction (AMI) in younger adults represents a distinct clinical entity with biological features that may differ from those observed in later-onset disease. We aimed to characterize age-dependent clinical, inflammatory, lipid, and echocardiographic phenotypes and to explore their association with in-hospital adverse clinical events. **Methods:** In this prospective, single-center, age-stratified observational study conducted between 20 January 2024 and 20 January 2025, 90 patients with AMI undergoing successful percutaneous coronary intervention (PCI) were selected from 1051 AMI admissions: 30 aged 25–44 years and 60 aged ≥45 years. Admission biomarkers (including C-reactive protein, leukocyte-derived indices, and lipid parameters) and echocardiographic variables (left ventricular ejection fraction and global longitudinal strain) were assessed, and exploratory receiver operating characteristic analyses were performed for in-hospital adverse clinical events. **Results:** Younger patients demonstrated a predominantly atherogenic profile, with higher smoking prevalence (63.3% vs. 13.3%; *p* < 0.001) and markedly higher lipoprotein(a) concentrations (76.18 ± 10.10 vs. 17.61 ± 11.42 mg/dL; *p* < 0.001). Older patients exhibited higher inflammatory indices (NLR and SII), higher hs-cTnI and NT-proBNP concentrations, and worse ventricular function. STEMI predominated in both age groups (83.3% in each), and infarct localization did not differ significantly between younger and older patients. The rate of in-hospital adverse clinical events was numerically higher in older patients (18.3% vs. 10.0%), but this difference was not statistically significant. In exploratory ROC analyses, CRP (AUC = 0.874) and LVEF (AUC = 0.868) showed the highest discriminatory performance. **Conclusions:** AMI appears to manifest through age-related baseline phenotypes: a more atherogenic/thrombotic profile in younger patients and a more inflammatory/ventricular dysfunction profile in older patients. CRP and LVEF emerged as the strongest exploratory discriminators of in-hospital adverse clinical events, but these findings require validation in larger cohorts.

## 1. Introduction

Cardiovascular disease (CVD) is a major cause of premature mortality around the world. About one in three deaths are caused by CVD, and in Europe, ischemic heart disease kills about 1.8 million people each year. Worldwide, the definition of premature coronary heart disease continues to be ambiguous. The World Health Organization defines young adulthood as ages 25 to 44; however, the age threshold for myocardial infarction (MI) in this demographic varies significantly among studies, impacting comparability and resulting in considerable discrepancies in reported incidence estimates across diverse populations and diagnostic criteria [1].

Over the past few decades, the number of young adults who develop premature acute myocardial infarction (AMI) has risen. About two-thirds of these cases are ST-segment elevation myocardial infarction (STEMI). Younger patients often show unusual symptoms compared to older adults, and they may have worse clinical outcomes given that there is not sufficient age-specific evidence and management guidelines. Younger patients, on the other hand, often develop acute events when vulnerable, lipid-rich plaques rupture in the middle of significant vascular inflammation, sometimes even without any of the common risk factors. This contrasts with older people who have diffuse atherosclerosis and multiple comorbidities [2,3,4,5].

**Figure 1:**
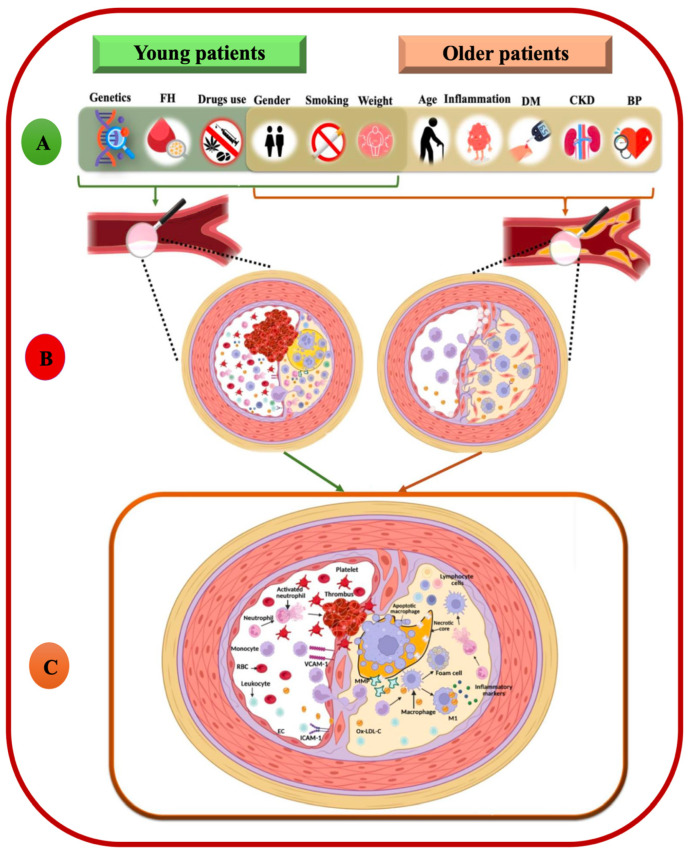
Comparative Pathophysiology of AMI in young vs. older patients: Inflammation as a Mechanism for Plaque Instability and Generation of Biomarkers. (**A**). Risk factor for atherosclerosis. Atherosclerosis in young patients is primarily driven by acute inflammation and plaque rupture due to toxic–metabolic factors like smoking, whereas in older individuals, it results from “inflamm-aging” and chronic comorbidities, leading to stiff, fibro-calcific arterial lesions. (**B**). Morphological substrate of AMI: Premature AMI is typically driven by the rupture of lipid-rich, thin-cap plaques in relatively healthy vessels, whereas traditional AMI in older patients involves the occlusion of diffusely calcified, chronically stenotic arteries through either plaque rupture or superficial erosion. (**C**). Common pathway: Atherosclerosis is a complex immune-driven inflammatory process where the interaction between endothelial cells, cholesterol-laden macrophages (foam cells), neutrophils, and lymphocytes leads to the progressive formation, destabilization, and potential rupture of arterial plaques. This image was created with BioRender (https://biorender.com).

Early biomarker evaluation post-acute myocardial infarction (AMI) may enhance risk stratification for major adverse cardiovascular events (MACE) by recognizing at-risk patients, thereby facilitating customized treatment approaches. A significant amount of research focuses on biomarkers from the harmed myocardium, but atherosclerosis is a complex mechanism that involves the immune system and interactions between different immune cells, such as neutrophils, monocytes, lymphocytes, and platelets (Figure 1). Neutrophils, as important parts of the innate immune system, contribute to breaking down plaque, while monocytes make inflammation worse and weaken the plaque structure. Platelets increase inflammation in vascular structures and the formation of blood clots. On the other hand, lymphocytes, especially regulatory T cells, have protective anti-inflammatory effects. Lymphopenia during acute myocardial infarction correlates with poor prognosis due to decreased control of systemic inflammation [5,6,7,8,9]. Recent studies have examined the etiology, risk factors, and the role of novel biomarkers in influencing outcomes for younger patients with myocardial infarction (MI) [10,11].

Composite hematologic indices, including the neutrophil-to-lymphocyte ratio (NLR), monocyte-to-lymphocyte ratio (MLR), platelet-to-lymphocyte ratio (PLR), and systemic immune–inflammation index (SII), provide an extensive depiction of the inflammatory-thrombotic milieu, indicating the equilibrium between pro-inflammatory innate immunity and the adaptive immune response. The existing evidence concerning the prognostic relevance of these indices is scarce and contradictory. Although an optimal biomarker panel for myocardial ischemia has not yet been established, recent studies suggest that multi-biomarker approaches may improve prognostic precision. Traditional biomarkers are well established in clinical practice, whereas non-traditional indices demonstrate potential as independent predictors of the no-reflow phenomenon, MACE, and mortality, both short- and long-term. Nonetheless, insufficient evidence persists regarding their efficacy in guiding treatment, particularly for young adults predisposed to recurrent coronary events [1,4,12].

The aim of the present study was to evaluate the association between baseline inflammatory biomarkers, lipid parameters, and echocardiographic indices and in-hospital adverse clinical events in young patients presenting with AMI (STEMI and NSTEMI), and to compare these findings between younger and older adults.

## 2. Materials and Methods

### 2.1. Study Design, Setting and Patient Selection

We conducted a prospective, single-center, age-stratified observational study at the Surveillance and Advanced Treatment Unit for Critical Cardiac Patients, “Prof. Dr. George I.M. Georgescu” Institute of Cardiovascular Diseases, Iași, Romania. Consecutive eligible patients were screened between 20 January 2024 and 20 January 2025. From 1051 admissions for AMI, 90 patients with AMI who underwent successful PCI met the eligibility criteria and were included. Participants were stratified by age into a younger group (25–44 years, *n* = 30) and an older comparator group (≥45 years, *n* = 60). MI was confirmed according to the Fourth Universal Definition of Myocardial Infarction, and STEMI/NSTEMI (non-ST-elevation myocardial infarction) classification was based on electrocardiographic criteria. New-onset left bundle branch block meeting Sgarbossa criteria was categorized as STEMI [13,14].

The study protocol was approved by the Ethics Committee of “Grigore T. Popa” University of Medicine and Pharmacy of Iași on 5 December 2023 (approval number 365/2023). All participants provided written informed consent before enrollment.

To provide a contemporaneous age comparator, the older group (≥45 years) was assembled as a prespecified 1:2 sample relative to the younger group during the same recruitment period. The younger group included all consecutive eligible patients aged 25–44 years who provided written informed consent. By contrast, the older group was sampled from eligible patients aged ≥45 years enrolled during the same time interval in order to create an age-stratified comparator cohort of manageable size for detailed biomarker and echocardiographic analysis.

Selection into the older comparator group was not based on left ventricular ejection fraction, outcome status, or severity of presentation. LVEF was assessed after enrollment as a study variable in all included participants and was not used to determine eligibility. Because the comparator group was sampled rather than exhaustively enrolled, between-group analyses were performed as unmatched comparisons, and the possibility of selection bias is acknowledged as a study limitation.

**Figure 2:**
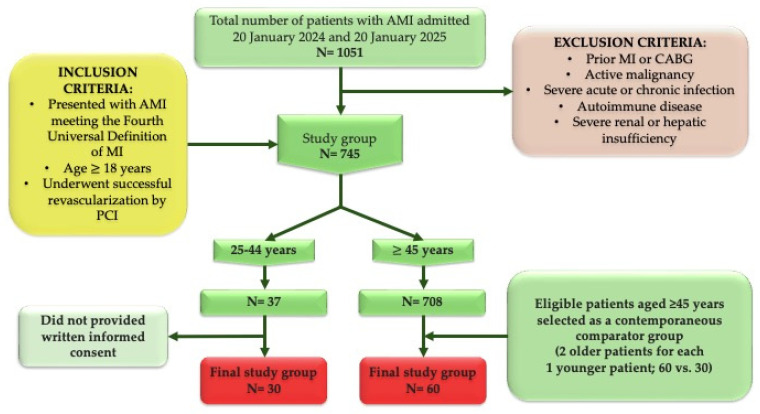
Study flow. AMI, acute myocardial infarction; CABG, coronary artery bypass grafting; MI, myocardial infarction; PCI, percutaneous coronary intervention.

Patients were eligible if they (i) presented with AMI meeting the Fourth Universal Definition of MI, (ii) were aged 25–44 years (young group) or ≥45 years (older group), (iii) underwent successful revascularization by PCI, and (iv) provided written informed consent. Patients were excluded if they had a prior MI or coronary artery bypass grafting (CABG), active malignancy, severe acute or chronic infection, autoimmune disease, or severe renal or hepatic insufficiency, given the potential for these conditions to independently influence systemic inflammation and the hematologic inflammatory indices under investigation (Figure 2).

### 2.2. Clinical Data and Laboratory Measurements

Demographic characteristics, cardiovascular history, and admission clinical variables were recorded at presentation. Peripheral venous blood was collected within 24 h of admission and prior to PCI. Standard hematologic and biochemical parameters were measured using automated analyzers in the institutional laboratory.

Inflammation-related composite indices were calculated as: NLR = neutrophils-to- lymphocytes; PLR = platelets-to-lymphocytes; MLR = monocytes-to-lymphocytes; SII = (platelets × neutrophils)-to-lymphocytes.

All participants underwent transthoracic echocardiography during the index hospitalization using a commercially available ultrasound system. Standard two-dimensional and Doppler measurements were acquired in accordance with current ASE/EACVI recommendations. Left ventricular systolic function was assessed by the biplane Simpson method to obtain LVEF. GLS was assessed by speckle-tracking echocardiography from apical views using General Electric Healthcare, Vivid E95, analyzed by Echo-PAC software (version 13.1.1) as a complementary marker of myocardial systolic dysfunction. Analyses were performed offline by experienced echocardiographers blinded to age-group allocation, and all strain analyses were conducted on a single-vendor platform to ensure internal standardization.

Coronary angiography and PCI were performed according to contemporary guideline-directed care [13,14]. Coronary anatomy, culprit lesion identification, and revascularization strategy were recorded for the present analysis.

The primary outcome was in-hospital adverse clinical events during the index hospitalization, defined as a composite of cardiovascular death, recurrent myocardial infarction, clinically significant arrhythmias requiring pharmacological or electrical treatment, cardiogenic shock, and acute heart failure requiring escalation of therapy. No post-discharge follow-up was included in the present analysis.

### 2.3. Statistical Analysis

Statistical analyses were performed using IBM SPSS Statistics (version 26.0). Data distribution was assessed using the Kolmogorov–Smirnov test. Continuous variables are presented as mean ± standard deviation (SD) for approximately normally distributed data or as median (interquartile range) for non-normally distributed data, whereas categorical variables are presented as counts and percentages. Between-group comparisons (younger: 25–44 years vs. older: ≥45 years) were performed using the independent-samples t test for normally distributed continuous variables and the Mann–Whitney U test for skewed variables. Categorical variables were compared using the χ^2^ test or Fisher’s exact test, as appropriate. The occurrence of in-hospital adverse clinical events was analyzed descriptively and through univariable comparisons between patients with and without events. Given the limited number of outcome events, no multivariable regression modeling was performed, and the study was not designed to support the identification of independent predictors. Receiver operating characteristic (ROC) curve analyses were performed as exploratory analyses to assess the discriminatory performance of selected baseline variables for in-hospital adverse clinical events, including CRP, NLR, SII, LVEF, and GLS. The area under the curve (AUC), 95% confidence intervals, and optimal cut-off values based on the Youden index were reported. These ROC analyses were considered hypothesis-generating and were not intended to establish clinical prediction models. A two-sided *p* value < 0.05 was considered statistically significant.

## 3. Results

### 3.1. Baseline Demographic and Clinical Characteristics

From 1051 consecutive AMI admissions screened during the study period, 90 patients met the study criteria and were included in the final analysis: 30 younger patients (25–44 years), representing all eligible consented young patients during the recruitment period, and 60 older patients (≥45 years), constituting a contemporaneous comparator sample selected in a prespecified 1:2 ratio.

Sex distribution, alcohol use, hypertension, and diabetes mellitus did not differ significantly between groups. Diabetes mellitus was present in 4 of 30 younger patients and in 8 of 60 older patients (13.3% vs. 13.3%, *p* = 1.000). Active smoking was substantially more frequent among younger patients (63.3% vs. 13.3%; *p* < 0.001), whereas dyslipidemia was highly prevalent in both strata and remained more frequent in the younger group (100% vs. 86.7%; *p* = 0.048).

At admission, body mass index and diastolic blood pressure were similar between groups. Younger patients presented with higher heart rate (98.96 ± 11.56 vs. 73.87 ± 11.75 bpm; *p* < 0.001), whereas systolic blood pressure was higher in the older group (146.28 ± 14.58 vs. 132.60 ± 15.65 mmHg; *p* < 0.001). Time from hospital presentation to PCI, reported in hours for the mixed STEMI/NSTEMI cohort, was shorter in the younger group (11.83 ± 10.43 vs. 18.75 ± 18.12 h; *p* = 0.024).

### 3.2. Laboratory Profile and Inflammation-Related Indices

Markers of myocardial injury and hemodynamic stress were higher in older patients, including hs-cTnI (2757.5 ± 89.57 vs. 914.5 ± 65.87 ng/L; *p* < 0.001) and NT-proBNP (186.29 ± 21.64 vs. 129.15 ± 11.56 pg/mL; *p* < 0.001).

A more pronounced atherogenic lipid profile was observed in the young group, with higher lipoprotein(a) (76.18 ± 10.10 vs. 17.61 ± 11.42 mg/dL; *p* < 0.001), total cholesterol (238.73 ± 55.67 vs. 209.00 ± 62.84 mg/dL; *p* = 0.026), LDL cholesterol (172.40 ± 46.32 vs. 140.70 ± 45.95 mg/dL; *p* = 0.003), and monocyte-to-HDL cholesterol ratio (16.76 ± 8.5 vs. 11.98 ± 9.1; *p* = 0.017). Mean platelet volume was higher in the older group (8.80 ± 0.99 vs. 8.38 ± 0.75; *p* = 0.028).

Distinct inflammatory signatures were observed across the age spectrum. Compared with younger patients, the older group exhibited higher NLR (5.60 ± 0.83 vs. 4.81 ± 0.83; *p* < 0.001) and SII (143 ± 17.37 vs. 108 ± 13.61 × 10^9^ cells/L; *p* < 0.001), whereas the younger group showed higher PLR (171.72 ± 83.31 vs. 124.97 ± 61.23; *p* = 0.009). MLR did not differ significantly between age strata (Table 1).

### 3.3. Echocardiographic and Angiographic Characteristics

Left ventricular volumes were comparable between age strata. However, systolic function was relatively preserved in younger patients, with higher LVEF (48.83 ± 8.59% vs. 39.97 ± 8.95%; *p* < 0.001), more negative GLS values (−15.44 ± 3.84% vs. −12.45 ± 3.15%; *p* < 0.001), and higher LVOT VTI (18.02 ± 3.25 vs. 15.23 ± 4.12 cm; *p* < 0.001).

Among diastolic indices, E/A ratio was higher in younger patients (1.06 ± 0.29 vs. 0.90 ± 0.41; *p* = 0.036). Average E/E′ tended to be higher in the older group (9.41 ± 2.95 vs. 8.22 ± 2.69), but this difference did not reach statistical significance (*p* = 0.060). LAVI also did not differ significantly between groups.

The clinical presentation of the index MI was comparable between groups. STEMI represented 83.3% of cases in both the younger and older groups (25/30 vs. 50/60), while NSTEMI accounted for 16.7% in each group (5/30 vs. 10/60). Infarct localization also did not differ significantly between age (overall, *p* = 0.357). Among younger patients, anterior and inferior/infero-lateral infarction were the most frequent patterns (50% and 33.3%), whereas anterior infarction predominated in older patients (55%).

Coronary anatomy was broadly comparable between groups. One-vessel disease was the predominant pattern in both groups (70.0% in younger vs. 83.4% in older patients; overall *p* = 0.161), and the distribution of culprit vessels did not differ significantly between age groups (overall *p* = 0.820) (Table 2).

### 3.4. In-Hospital Adverse Clinical Events

During the index hospitalization, 14 patients experienced in-hospital adverse clinical events. The outcome components were cardiovascular death (*n* = 4), clinically significant arrhythmias requiring pharmacological or electrical treatment (*n* = 7), and cardiogenic shock (*n* = 3). No recurrent MI or acute heart failure requiring escalation of therapy was recorded during the index hospitalization. The crude event rate was numerically higher in the older group than in the younger group (18.3% vs. 10.0%), but this between-group difference was not statistically significant (Fisher’s exact *p* = 0.370) (Table 3).

### 3.5. ROC Analyses for In-Hospital Adverse Clinical Events

**Figure 3:**
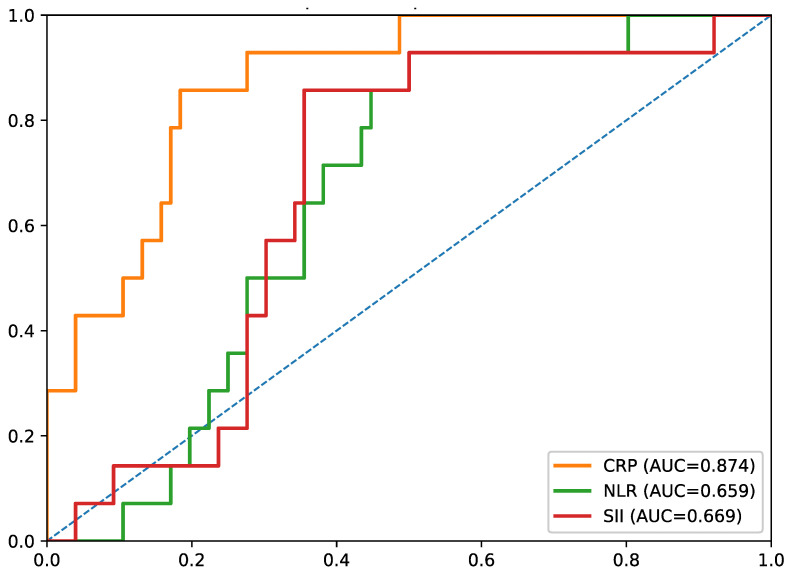
Receiver operating characteristic curves of CRP, NLR, and SII for prediction of in-hospital adverse clinical events. CRP, C-reactive protein; NLR, neutrophil-to-lymphocyte ratio; SII, systemic immune–inflammation index.

Exploratory ROC analyses were performed for in-hospital adverse clinical events. Among inflammatory biomarkers, CRP showed the highest discriminatory performance (AUC = 0.874), whereas NLR (AUC = 0.659) and SII (AUC = 0.669) showed only moderate discrimination (Figure 3).

**Figure 4:**
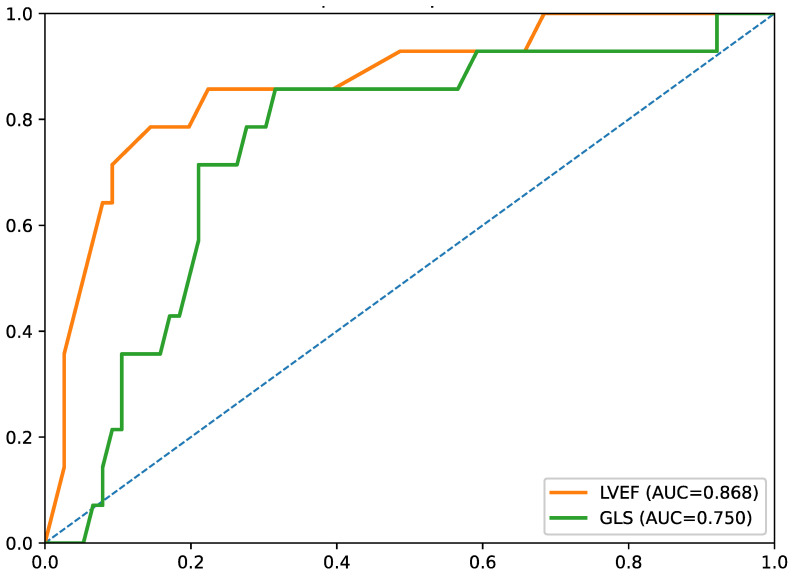
Receiver operating characteristic curves of LVEF and LV GLS for prediction of in-hospital adverse clinical events. LVEF, left ventricular ejection fraction; LV GLS, left ventricular global longitudinal strain.

Among echocardiographic parameters, LVEF demonstrated the strongest discrimination (AUC = 0.868), while GLS showed moderate discrimination (AUC = 0.750) (Figure 4).

Given the small sample size and the limited number of in-hospital events (*n* = 14), these ROC findings should be interpreted with considerable caution. In particular, the relatively high AUC values observed for CRP and LVEF may reflect optimistic estimates in a small exploratory dataset and should be regarded as hypothesis-generating rather than confirmatory.

## 4. Discussion

The clinical presentation of AMI in younger adults is still a distinct problem, but new evidence shows that age-related differences are mostly due to differences in baseline biological profiles rather than differences in coronary anatomy. As shown in previous studies, younger patients in this cohort had more exposure to smoking and less favorable lipid profiles, but their ventricular function continued to be better preserved. Conversely, older patients demonstrated the expected patterns of greater myocardial injury, higher inflammatory markers, and more pronounced systolic impairment. The notably high prevalence of active smoking among younger patients underscores its well-documented role as a primary driver of premature AMI. This observation is entirely consistent with prior cohorts, which identify smoking as the dominant modifiable risk factor in young-onset myocardial infarction. In these instances, the pathology typically arises from the synergy between tobacco use, dyslipidemia, and genetic susceptibility, rather than the cumulative burden of long-term comorbidities seen in older populations [15,16,17,18,19,20,21].

The lipid phenotype in the younger group is also clinically relevant Notably, the most pronounced difference observed was the markedly higher Lp(a) concentration in younger patients (approximately four-fold greater than in the older group), consistent with the established role of Lp(a) as an independent, genetically mediated driver of premature atherothrombosis. Research shows that elevated Lp(a) levels are closely associated with myocardial infarction (MI) and aortic valve stenosis (AVS), while showing weaker connections to ischemic stroke and overall mortality. Clinically significant Lp(a) levels exceed 50 mg/dL, largely influenced by genetics. Levels above 30–50 mg/dL triple the risk of coronary artery disease, particularly in the absence of other risk factors [22,23].

Lp(a) measurement is advised for all adults at least once, especially for those with familial hypercholesterolemia, early atherosclerotic cardiovascular disease, or a family history of high Lp(a) levels. Current data do not support the notion that Lp(a) enhances risk prediction beyond standard scores. However, high Lp(a) levels (>47 mg/dL) can help reclassify the risk of MI, which could put people in a higher risk group than what the SCORE2 or SCORE2-OP algorithms say. This could change LDL-C goals and strategies for lowering lipids. The effects of Lp(a) reduction on cardiovascular disease progression are still uncertain, necessitating early risk management and proactive LDL-C lowering. Currently, specific Lp(a)-lowering medications are undergoing randomized clinical trials. These include injectable RNA-based therapies targeting apolipoprotein(a) production in the hepatocyte, which can lower Lp(a) concentration by 80–98%. Additionally, an oral small molecule inhibitor and a small interfering RNA that can significantly reduce Lp(a) are also being investigated [15,22,23,24,25,26,27,28,29]. The markedly elevated lipoprotein(a) levels observed in younger patients are clinically important, as they suggest a stronger inherited atherothrombotic burden in premature AMI. This finding supports the routine assessment of Lp(a) in younger patients presenting with myocardial infarction, particularly because elevated Lp(a) may identify individuals with residual cardiovascular risk that is not captured by conventional lipid parameters alone. In this context, the early recognition of high Lp(a) may have implications for long-term risk stratification, family screening, and the intensification of preventive strategies, while also gaining relevance in view of emerging Lp(a)-targeted therapies.

Older patients exhibited higher hs-cTnI and NT-proBNP, a pattern consistent with larger acute injury burden and/or lower myocardial reserve. The concordant reduction in LVEF, less negative GLS, and lower LVOT VTI further suggests that the older group entered the acute event with less functional reserve and greater vulnerability to hemodynamic deterioration. The integration of GLS alongside LVEF provides a more nuanced assessment of myocardial mechanics, potentially identifying subtle systolic impairment that conventional volumetry might overlook during the acute phase of MI. Furthermore, the observation of higher SII levels in older patients aligns with the concept of ‘inflamm-aging,’ suggesting that this composite index serves as a pragmatic proxy for the intensified innate immune response often seen in later-onset disease [18,19,20,30].

Of note, the difference in time from presentation to PCI should be interpreted cautiously because the cohort included both STEMI and NSTEMI and the variable was reported in hours rather than as a strict STEMI door-to-balloon metric. Even so, the direction of effect is compatible with previous reports showing earlier presentation or faster pathway activation in younger adults [13,14].

Inflammation-related indices showed an age-dependent pattern. Higher NLR and SII in older patients are consistent with a more pronounced systemic inflammatory response during AMI, whereas higher PLR in younger patients may reflect a relatively stronger platelet-related thrombotic signal [31,32,33,34,35,36,37,38,39].

From a biological perspective, an elevated Neutrophil-to-Lymphocyte Ratio (NLR) indicates significant imbalance contributing to atherosclerotic plaque instability and myocardial infarction through several mechanisms. First, neutrophils play an aggressive role in inflammation and plaque degradation by infiltrating the vascular wall and the plaque itself, releasing pro-inflammatory mediators like IL-1 and IL-6, reactive oxygen species, and various proteolytic enzymes. This leads to plaque instability as these substances degrade collagen and the extracellular matrix, thinning the protective fibrous cap and turning the plaque into a vulnerable structure prone to rupture. Second, the formation of Neutrophil Extracellular Traps (NETs) occurs as activated neutrophils release decondensed DNA filaments that capture other leukocytes and initiate platelet activation, thus accelerating thrombus formation upon plaque rupture [37,40,41,42]. Activated platelets, which are usually referred to for their role in coagulation, are important for promoting atherosclerotic inflammation due to their ability to release vasoactive and pro-inflammatory mediators. They bind to receptors on the vascular endothelium, such as P-selectin and GPIIb/IIIa, which helps monocytes and leukocytes migrate. This process enhances inflammation and plaque stability. When atherosclerotic plaques break, they release procoagulant substances, resulting in increased platelet reactivity and causing thrombus formation to occur promptly. This blocks blood flow and causes myocardial ischemia. Platelet aggregation complicates the situation further by preventing the restoration of circulation after repair, leading to the “no-reflow” phenomenon [38,43,44,45].

Lastly, lymphocytes, particularly regulatory T cells, typically suppress inflammation and stabilize plaques. However, during ischemic events or systemic stress, the release of stress hormones like catecholamines and cortisol hinders lymphocyte function by inducing apoptosis, leading to a decrease in lymphocyte count. This deficiency results in an ineffective immune and anti-inflammatory response, enabling unchecked neutrophil action and platelets and further promoting plaque instability and risk of myocardial infarction [44,45].

In conclusion, high PLR and NLR signify platelet and neutrophil hyper-reactivity and lymphocyte depletion, promoting inflammation, plaque rupture, and thrombus formation, leading to coronary vessel occlusion and subsequent myocardial necrosis. After the infarction, increased neutrophils exacerbate cardiac damage through free radicals and proteases, intensifying reperfusion injury. Moreover, an elevated SII precisely captures the underlying mechanism of myocardial infarction: a state where aggressive thrombo-inflammation, fueled by the alliance between plaque-rupturing neutrophils and clot-forming platelets, operates completely unchecked due to the suppression of adaptive immunity (lymphocyte depletion).

The outcome analysis also requires careful interpretation. Although the crude in-hospital adverse-event rate was higher in older patients, the between-group difference was not statistically significant after recalculation from the reported event counts. Accordingly, age group alone should not be overinterpreted as an independent determinant of early in-hospital events in this dataset.

A central observation of the study is that CRP and LVEF had the highest AUCs in exploratory ROC analyses. This suggests that, within the acute in-hospital phase, a general marker of inflammatory activation and a direct measure of ventricular dysfunction may capture short-term clinical vulnerability better than leukocyte-derived ratios alone [46,47]. However, this observation must be interpreted cautiously, as only 14 in-hospital events were recorded in a cohort of 90 patients. Under these conditions, the apparent discriminatory performance of CRP and LVEF may be unstable and potentially overestimated, and these results should not be interpreted as evidence of robust predictive utility.

At the same time, the ROC results must be viewed as hypothesis-generating. Only 14 outcome events were recorded, which limits precision and increases the risk of optimistic estimates. In this context, the absence of a stable multivariable model is appropriate, and the pre-sent results cannot establish incremental predictive value over validated clinical risk scores or support firm cut-off-based clinical decision-making [48,49,50,51].

Overall, our findings should be interpreted primarily as a confirmation and refinement of previously described age-related patterns in AMI rather than as evidence of a fundamentally novel clinical phenotype. The main contribution of the present study is the integrated characterization of inflammatory, lipid, and echocardiographic features within the same exploratory cohort. In younger patients, the combination of smoking burden and atherogenic lipid abnormalities identifies a preventive target profile, whereas in older patients, inflammatory activation and ventricular dysfunction appear more closely linked to early in-hospital vulnerability. These findings warrant validation in larger multicenter cohorts before they are translated into management algorithms.

## 5. Clinical Implications and Future Directions

These findings support an age-informed approach to bedside risk assessment in AMI. In younger patients, emphasis should be placed on smoking cessation, comprehensive lipid profiling, and recognition of markedly elevated Lp(a) as a possible marker of inherited residual risk. In older patients, early assessment of inflammatory burden and ventricular function may help identify patients requiring closer in-hospital monitoring. Our findings underscore that AMI in patients under 45 is not merely an early version of the disease seen in older adults but supports the hypothesis of a potent atherogenic and thrombotic substrate, highlighted by significantly elevated lipoprotein(a) levels and a higher smoking prevalence. This necessitates a shift toward age-tailored secondary prevention strategies that prioritize aggressive lipid management and smoking cessation in the younger population of Eastern Europe. Future studies should validate combined biomarker and echocardiographic models in larger multicenter populations and test their added value beyond established clinical risk tools.

## 6. Strengths and Limitations

This study should be viewed as a preliminary, hypothesis-generating analysis designed to delineate age-related differences in patients hospitalized with ACS and to identify clinically relevant inflammatory, lipid, and echocardiographic patterns for subsequent validation.

Several limitations should be considered when interpreting these findings. First, the single-center design inherently limits external validity and may constrain generalizability beyond the studied population and healthcare setting. Second, although the younger cohort consisted of consecutive eligible patients, the older comparator group was assembled in a prespecified 1:2 ratio rather than through exhaustive consecutive enrollment, which may have introduced selection bias and reduced representativeness. Third, the modest sample size and low number of in-hospital events limited statistical power and reduced the precision of the exploratory ROC analyses; consequently, the observed discriminatory performance of CRP and LVEF should be interpreted with appropriate caution. Fourth, the inclusion of both STEMI and NSTEMI patients introduced clinical heterogeneity. Finally, because the analysis was restricted to the index hospitalization, the present study does not provide insight into post-discharge or long-term outcomes.

## 7. Conclusions

This prospective age-stratified study suggests that AMI is associated with distinct baseline phenotypes across age groups. Younger patients exhibited a predominantly atherogenic profile, characterized by heavier smoking exposure, higher lipoprotein(a) levels, and higher PLR, together with relatively preserved ventricular function. In contrast, older patients showed greater myocardial injury, higher NLR and SII values, and more impaired systolic function. Among the baseline variables assessed during the index hospitalization, CRP and LVEF demonstrated the highest discriminatory performance in exploratory analyses of in-hospital adverse clinical events. These findings support an age-informed interpretation of AMI presentation and early in-hospital risk; however, they should be regarded as descriptive and hypothesis-generating until validated in larger multicenter cohorts.

## Figures and Tables

**Table 1:** Patient characteristics at baseline.

Variable	Age Group < 45 Years (*n* = 30)	Age Group ≥ 45 Years (*n* = 60)	*p*-Value
Demographic characteristics and cardiovascular risk factors			
Age, mean ± SD	40.63 ± 4.04	59.83 ± 9.54	<0.001
Female, *n* (%)	12 (40%)	30 (50%)	0.502
Smoking, *n* (%)	19 (63.3%)	8 (13.3%)	<0.001
Alcohol user, *n* (%)	7 (23.3%)	17 (28.3%)	0.801
Hypertension, *n* (%)	3 (10%)	11 (19%)	0.370
Dyslipidemia, *n* (%)	30 (100%)	52 (86.7%)	0.048
Admission hemodynamics			
BMI (kg/m^2^), mean ± SD	27.83 ± 5.24	28.02 ± 3.69	0.860
Heart rate (bpm), mean ± SD	98.96 ± 11.56	73.87 ± 11.75	<0.001
SBP (mmHg), mean ± SD	132.60 ± 15.65	146.28 ± 14.58	<0.001
DBP (mmHg), mean ± SD	85.52 ± 10.58	82.19 ± 10.15	0.159
Time to PCI (hours)	11.83 ± 10.43	18.75 ± 18.12	0.024
Biological profile			
NT-proBNP (pg/mL), mean ± SD	129.15 ± 11.56	186.29 ± 21.64	<0.001
hs-cTnI (ng/L)	914.5 ± 65.87	2757.5 ± 89.57	<0.001
Lipoprotein (a) (mg/dL)	76.18 ± 10.10	17.61 ± 11.42	<0.001
Total cholesterol (mg/dL), mean ± SD	238.73 ± 55.67	209.00 ± 62.84	0.026
LDL cholesterol (mg/dL), mean ± SD	172.40 ± 46.32	140.70 ± 45.95	0.003
HDL cholesterol (mg/dL), mean ± SD	40.68 ± 11.11	39.93 ± 9.90	0.756
MPV, mean ± SD	8.38 ± 0.75	8.80 ± 0.99	0.028
Monocytes/HDL cholesterol	16.76 ± 8.5	11.98 ± 9.1	0.017
Neutrophils/Lymphocytes (NLR)	4.81 ± 0.83	5.60 ± 0.83	<0.001
Monocytes/Lymphocytes (MLR)	0.41 ± 0.33	0.44 ± 0.27	0.668
Platelets/Lymphocytes (PLR)	171.72 ± 83.31	124.97 ± 61.23	0.009
SII (×10^9^ cells/L)	108 ± 13.61	143 ± 17.37	<0.001

BMI, body mass index; DBP, diastolic blood pressure; hs-cTnI, High-sensitivity cardiac troponin I; HDL cholesterol, High-Density Lipoprotein cholesterol; LDL cholesterol, Low-Density Lipoprotein cholesterol; MPV, Mean platelet volume; NT-proBNP, N-terminal pro B-type natriuretic peptide; PCI, percutaneous coronary intervention; SBP, systolic blood pressure; SD, standard deviation.

**Table 2:** Echocardiographic and coronary angiography characteristics at baseline.

Variable		Age Group < 45 Years (*n* = 30)	Age Group ≥ 45 Years (*n* = 60)	*p*-Value
Echocardiographic characteristics				
LVEDV (mL)		119.77 ± 22.75	119.20 ± 21.79	0.910
LVESV (mL)		64.93 ± 16.06	68.00 ± 18.57	0.421
LVEF (%)		48.83 ± 8.59	39.97 ± 8.95	<0.001
VTI LVOT (cm)		18.02 ± 3.25	15.23 ± 4.12	<0.001
E/A		1.06 ± 0.29	0.90 ± 0.41	0.036
E/E’ (average)		8.22 ± 2.69	9.41 ± 2.95	0.060
LV GLS (%)		−15.44 ± 3.84	−12.45 ± 3.15	<0.001
LAVI (mL/m^2^)		46.46 ± 17.59	53.45 ± 14.52	0.066
Index MI presentation				
STEMI (%)		25 (83.3%)	50 (83.3%)	1.000
NSTEMI (%)		5 (16.7%)	10 (16.7%)	
Infarct localization, *n* (%)	Inferior/infero-lateral	10 (33.3%)	17 (28.3%)	0.357
	Postero-inferior/lateral	5 (16.7%)	10 (16.7%)	
	Anterior	15 (50%)	33 (55%)	
Coronary angiography characteristics				
Number of coronary artery disease	One-vessel disease	21 (70%)	50 (83.4%)	0.161
	Two-vessel disease	3 (10%)	6 (10%)	
	Three-vessel disease	6 (20%)	4 (6.6%)	
Culprit lesion LAD		21 (70%)	38 (63.4%)	0.820
Culprit lesion LCX		4 (13.3%)	10 (16.6%)	
Culprit lesion RCA		5 (16.7%)	12 (20%)	

LAD, left anterior descending coronary artery; LAVI, Left Atrial Indexed Volume; LCX, left circumflex coronary artery; LVEDV, Left Ventricular End-Diastolic Volume; LVEF, Left Ventricular Ejection Fraction; LVESV, Left Ventricular End-Systolic Volume; LV GLS, Left Ventricular Global Longitudinal Strain; MI, Myocardial infarction; NSTEMI, Non-ST-elevation myocardial infarction; RCA, right coronary artery; STEMI, ST-elevation myocardial infarction; VTI LVOT, Left Ventricular Outflow Tract Velocity Time Integral.

**Table 3:** Components of in-hospital adverse clinical events according to age group.

Variable	Age Group < 45 Years (*n* = 30)	Age Group ≥ 45 Years (*n* = 60)	*p*-Value
Any in-hospital adverse clinical event, *n* (%)	3 (10.0%)	11 (18.3%)	0.370
Clinically significant arrhythmias requiring treatment, *n* (%)	1 (3.3%)	6 (10%)	
Cardiogenic shock, *n* (%)	1 (3.3%)	2 (3.3%)	
Cardiovascular death, *n* (%)	1 (3.3%)	3 (5%)	

## Data Availability

De-identified study data are available on reasonable request from the corresponding author (larisa.anghel@umfiasi.ro). A justification for its further use should be provided.

## Abbreviations

The following abbreviations are used in this manuscript:

AMI	Acute myocardial infarction
AUC	Area under the curve
BMI	Body mass index
CABG	Coronary artery bypass grafting
CAD	Coronary artery disease
CRP	C-reactive protein
CVD	Cardiovascular disease
D2B	Door-to-balloon
DBP	Diastolic blood pressure
GLS	Global longitudinal strain
HDL-C	High-density lipoprotein cholesterol
HF	Heart Failure
hs-cTnI	High-sensitivity cardiac troponin I
LAD	Left anterior descending coronary artery
LAVI	Left Atrial Indexed Volume
LCX	Left circumflex coronary artery
LDL-C	Low-density lipoprotein cholesterol
Lp(a)	Lipoprotein(a)
LVEDV	Left Ventricular End-Diastolic Volume
LVEF	Left Ventricular Ejection Fraction
LVESV	Left Ventricular End-Systolic Volume
MACE	Major adverse cardiovascular events
MI	Myocardial infarction
MLR	Monocytes-to-Lymphocytes ratio
MPV	Mean platelet volume
NETs	Neutrophil extracellular traps
NLR	Neutrophils-to-Lymphocytes ratio
NSTEMI	Non-ST-elevation myocardial infarction
NT-proBNP	N-terminal pro B-type natriuretic peptide
OR	Odds ratio
PCI	Percutaneous coronary intervention
PLR	Platelets-to-Lymphocytes ratio
RCA	Right coronary artery
ROC	Receiver operating characteristic
SBP	Systolic blood pressure
SD	Standard deviation
SII	Systemic immune–inflammation index
STEMI	ST-elevation myocardial infarction
VTI LVOT	Left Ventricular Outflow Tract Velocity Time Integral
